# uPAR PET/CT for Prognostication and Response Assessment in Patients with Metastatic Castration-Resistant Prostate Cancer Undergoing Radium-223 Therapy: A Prospective Phase II Study

**DOI:** 10.3390/diagnostics11061087

**Published:** 2021-06-14

**Authors:** Marie Øbro Fosbøl, Jann Mortensen, Peter Meidahl Petersen, Annika Loft, Jacob Madsen, Andreas Kjaer

**Affiliations:** 1 Department of Clinical Physiology, Nuclear Medicine & PET, Copenhagen University Hospital, Rigshospitalet, 2100 Copenhagen, Denmark; marie.oebro.fosboel@regionh.dk (M.Ø.F.); jann.mortensen@regionh.dk (J.M.); annika.loft.jakobsen@regionh.dk (A.L.); Jacob.madsen@regionh.dk (J.M.); 2 Cluster for Molecular Imaging, Department of Biomedical Sciences and Department of Clinical Medicine, University of Copenhagen, 2200 Copenhagen, Denmark; 3 Department of Oncology, Copenhagen University Hospital, Rigshospitalet, 2100 Copenhagen, Denmark; peter.meidahl.petersen@regionh.dk

**Keywords:** urokinase-type plasminogen activator receptor (uPAR), prostate cancer, PET, Radium-223 therapy

## Abstract

The aim of this Phase II study was to investigate the potential for response assessment and prognostication of positron emission tomography (PET) using the ligand ^68^Ga-NOTA-AE105 targeting the urokinase-type plasminogen activator receptor (uPAR) in patients receiving Radium-223-dichloride therapy (^223^RaCl_2_). A combined whole-body uPAR PET and computed tomography (CT) was performed before initiation of ^223^RaCl_2_ and after two cycles of therapy. Standardized uptake value (SUV) in selected bone metastases was measured and the lesion with the highest SUV_max_ was considered the index lesion. Clinical outcomes were overall survival (OS), radiographic progression free survival (rPFS) and occurrence of symptomatic skeletal event (SSE). A total of 17 patients were included and 14 patients completed both baseline and follow-up uPAR-PET/CT. Baseline SUV_max_ of the index lesion was associated with OS; hazard ratio 2.51 (95% CI: 1.01–6.28, *p* = 0.05) per unit increase in SUV_max_. No association between changes in SUV_max_ from baseline to follow-up and OS, progression during therapy, or rPFS was found. Baseline SUV_max_ was a significant predictor of SSE with receiver operating characteristics (ROC) area under the curve (AUC) = 0.81 (95% CI: 0.58–1.00, *p* = 0.034). A cut-off for tumor SUV_max_ could be established with an odds ratio of 14.0 (95% CI: 1.14–172.6, *p* = 0.023) for occurrence of SSE within 12 months. Although based on a small number of patients, uPAR-PET SUV_max_ in bone metastases was predictive for OS and risk of SSE in mCRPC patients receiving ^223^RaCl_2_. However, a relatively low uptake of the uPAR ligand in bone metastases impedes visual evaluation and requires another modality for lesion delineation.

## 1. Introduction

Radium-223-dichloride (^223^RaCl_2_) is the first alpha-emitting radionuclide therapy with a documented effect on overall survival (OS) and time to symptomatic skeletal related event (SSE) in patients with bone metastases from metastatic castration-resistant prostate cancer (mCRPC) [1,2]. However, interim response monitoring poses a clinical challenge. Dynamics of serum prostate specific antigen (PSA) is not an appropriate marker of response as only a minority of patients exhibit PSA decline during therapy [3]. Bone targeted imaging with either ^99m^Tc-bisphophonate bone scintigraphy or ^18^F-fluoride Positron emission tomography (PET) presents inherent limitations due to the “flare phenomenon”, making these modalities less suitable for timely assessment in therapy with ^223^RaCl_2_ [4].

A promising imaging target in this context is the urokinase-type plasminogen activator receptor (uPAR). uPAR is a cell membrane protein involved in regulating extracellular matrix proteolysis. In several types of malignant tumors and tumor-associated stromal cells, uPAR is overexpressed and high expression levels are associated with invasive potential, metastasis and resistance to chemotherapy [5,6,7,8,9,10]. The degree of uPAR expression in prostate cancer specimens is associated with important clinicopathological prognostic features, such as pathological tumor stage, Gleason score, positive surgical margins, and shorter biochemical recurrence free survival [11,12].

Quantification of uPAR expression could be a promising method of response assessment by estimating changes in the invasive potential of malignant tissue during the course of therapy. Non-invasive evaluation of uPAR expression is possible with the use of the uPAR specific PET imaging radioligand ^68^Ga-NOTA-AE105. ^68^Ga-NOTA-AE105 PET has been validated in a phase I study including prostate cancer patients, where uptake of the ligand corresponded to uPAR expression in excised tissue [13]. Additionally, in a recent phase II PET/MRI trial in patients with prostate cancer, we found a clear correlation between tumor uptake of the ligand and Gleason score, which confirmed the ligand as an imaging marker of prostate cancer aggressiveness [14].

We therefore hypothesized that ^68^Ga-NOTA-AE105 could offer a possibility for predicting the therapeutic effect of ^223^RaCl_2_ by evaluating early changes in uPAR expression during therapy. The objective of the current study was to evaluate the potential of uPAR PET/CT for interim response assessment among mCRPC patients treated with ^223^RaCl_2_. A secondary aim was to explore the prognostic value of uPAR PET/CT in regard to overall survival (OS) and risk of SSE after therapy with ^223^RaCl_2_.

## 2. Materials and Methods

### 2.1. Study Design

Between December 2016 and March 2018, patients who were referred for ^223^RaCl_2_ at our institution were screened for eligibility. Inclusion criteria were: mCRPC, planned therapy with ^223^RaCl_2_, age ≥18 years and ability to give informed consent. Exclusion criteria were: Impaired communication skills or inability to understand study protocol, other known malignant disease or known allergy towards ^68^Ga-NOTA-AE105. Participants received ^223^RaCl_2_ therapy according to guidelines (55 kBq/kg intravenously every four weeks for up to six cycles). Clinical management of the patients was blinded for the uPAR PET results. Treatment was discontinued early in case of disease progression, unacceptable adverse events, declining performance status or by request from the patient. Patient characteristics collected at baseline included age, serum PSA, serum alkaline phosphatase, ECOG (Eastern Cooperative Oncology Group) performance status, and which systemic therapies for mCRPC the patients had received prior to ^223^RaCl_2_.

The study protocol was approved by the Danish Medicines Agency (EudraCT no: 2016-002184-34; trial sponsor: Rigshospitalet) and the Regional Scientific Ethical Committee (Protocol no. H-16036551). Signature of written informed consent was obtained from all patients. The study was registered in ClinicalTrials.gov (NCT02964988) and was performed in accordance with the recommendation for good clinical practice (GCP) including independent monitoring by the GCP unit of the Capital Region of Denmark.

### 2.2. PET/CT Acquisition

uPAR PET/CT was conducted within 30 days prior to first cycle of ^223^RaCl_2_ and within 30 days after the second cycle of therapy. The PET/CT scans were performed using an integrated PET/CT system (Biograph64 mCT; Siemens Medical Solutions, Erlangen, Germany) starting 20 min after intravenous administration of a fixed dose of approximately 200 MBq ^68^Ga-NOTA-AE105. Synthesis of the ligand was performed as described earlier [13]. Whole-body PET scans from mid femur to vertex were obtained in 3-dimensional mode, with an acquisition time of 4 min per bed position. Attenuation and scatter corrected PET data were reconstructed iteratively using a 3D ordered subset expectation maximization (OSEM) with four iterations, eight subsets. A diagnostic CT with 2-mm slice thickness, 100 kV, and a quality reference of 265 mAs modulated by the Care Dose 4D automatic exposure control system (Siemens Medical Solutions, Erlangen, Germany) was performed before the PET-scan.

### 2.3. Image Analysis

PET/CT scans were evaluated by two board certified specialists in nuclear medicine blinded to clinical outcomes. Volumes of interest (VOIs) were drawn on baseline uPAR PET/CT images, corresponding to up to three bone metastases, in each participant. Selection of lesions was guided by baseline bone imaging (bone scintigraphy or ^18^F-fluoride PET), where the most prominent lesions were identified based on size and the level of tracer uptake. The same lesions were identified and delineated on follow-up uPAR PET/CT. Uptake of ^68^Ga-NOTA-AE105 in normal tissue for reference was measured by placing spheric VOIs in the right liver lobe, thoracic aorta and psoas muscle.

Uptake of the uPAR ligand in the VOIs was parameterized as maximum standardized uptake value (SUV_max_). For each participant, the lesion with the highest SUV_max_ at baseline, corresponding to the highest level of uPAR expression, was registered as the index lesion.

### 2.4. Clinical Outcomes

After inclusion, patients were followed from date of first uPAR PET/CT up to 18 months after or date of death. OS was defined as the time from first ^223^RaCl_2_ cycle to death of any cause. The occurrence of first SSE defined as new pathological symptomatic fracture, use of external beam radiation therapy (EBRT) for bone pain, and spinal cord compression or tumor-related orthopedic intervention was registered within 12 months from date of first ^223^RaCl_2_ cycle. Routine imaging for response assessment consisted of bone scintigraphy and CT of thorax and abdomen after three and six cycles of ^223^RaCl_2_, respectively, performed by decision of the treating physician. Supplemental imaging procedures were performed in case of clinical suspicion of progression.

### 2.5. Statistical Analysis

Inter reader reliability of SUV-measurement was estimated using intraclass correlation coefficient (ICC). Kaplan–Meier statistics was used to calculate mean OS. Cox proportional hazards models were used to assess time-to-event outcomes: OS and radiographic progression free survival (rPFS) and association with SUV_max_. Univariate logistic regression analysis was used to investigate association between SUV_max_ and progression during therapy. The association between occurrence of SSE and index lesion SUV_max_ as a continuous variable was investigated by receiver operating characteristics (ROC) analysis. Based on the ROC analysis, a cut-off value of SUV_max_ was identified to achieve the highest accuracy in prediction of SSE. Pearson’s Chi-square test was used to calculate the odds ratio for the selected cutoff value in discriminating patients at risk of SSE. 

Statistical analyses were performed in SPSS, version 25 (IBM Corp., Armonk, NY, USA). Cases with missing data were excluded listwise. *p*  ≤  0.05 was considered statistically significant.

## 3. Results

**Figure 1:**
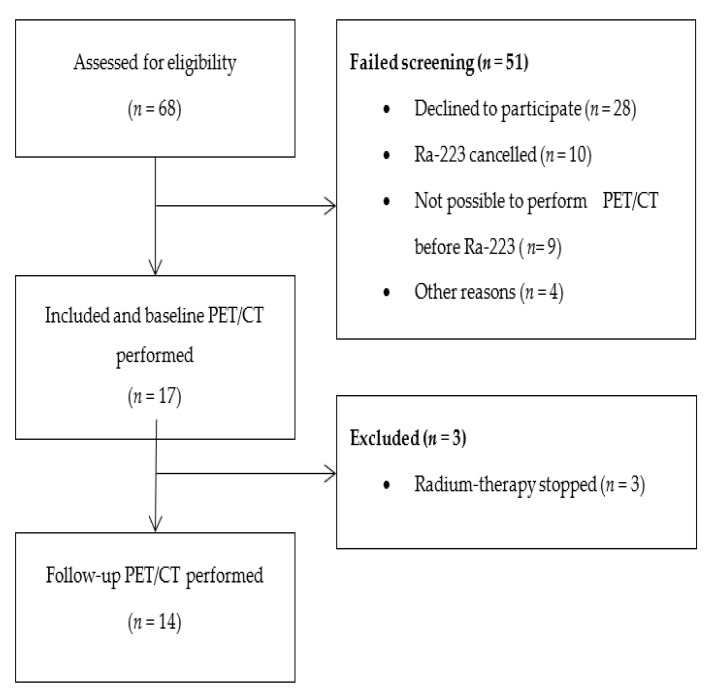
CONSORT diagram of the inclusion process.

Seventeen patients were included in the study (Figure 1). According to the study protocol, 43 patients were originally planned to be included, but due to challenges in the recruitment of participants, in part due to a decreasing number of patients referred to ^223^RaCl_2_ treatment at our institution, the study was ended with a lower number of patients. Baseline characteristics are displayed in Table 1.

Median time from baseline uPAR PET/CT to initiation of ^223^RaCl_2_ therapy was 4 days (range: 1–29). Patients received an intravenous dose of 200 MBq (median 201 MBq, range: 142–228 MBq) ^68^Ga-NOTA-AE105 per PET scan. This activity corresponded to an effective dose of approximately 3.1 mSv per PET scan according to dosimetry calculations from the phase I trial [13]. No adverse events or reactions related to the administration were observed. 

Three patients only received one cycle of ^223^RaCl_2_ and therefore did not undergo follow-up uPAR PET/CT. The reasons for discontinuation of therapy in these patients were deteriorating clinical condition (n = 2) and bone marrow suppression (n = 1). Median time from baseline uPAR PET/CT to follow-up scan was 56 days (range: 39–81 days).

### 3.1. PET/CT Results

At baseline uPAR PET/CT a total of 46 metastatic lesions were delineated by each observer and 37 lesions at follow-up PET/CT. The interrater variability in terms of ICC was 0.75 (95% CI: 0.6–0.9) for lesions at baseline and 0.85 (95% CI: 0.71–0.92) at follow-up PET/CT. SUV_max_ of the index lesion (i.e., lesion with the highest SUV_max_) at baseline and follow-up for each participant is displayed in Table 2.

**Figure 2:**
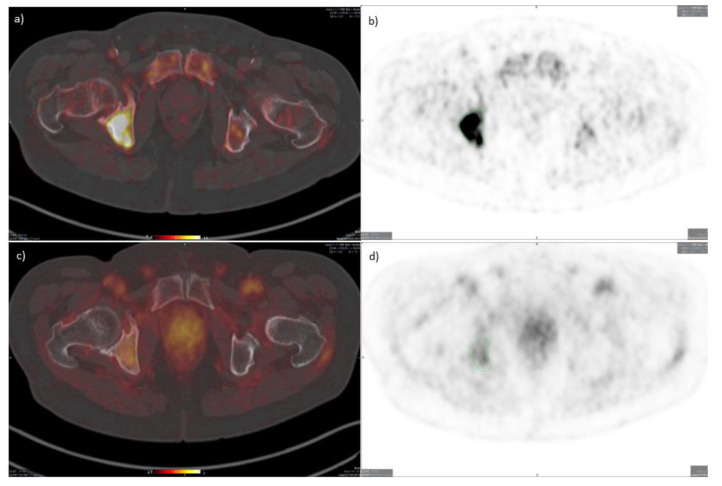
^18^F-fluoride and uPAR-PET/CT (patient 3) Transaxial fused images (left), PET images (right). (**a**,**b**): ^18^F-fluoride PET/CT. (**c**,**d**): uPAR-PET/CT (bottom row). Bone metastasis in the pelvis is delineated. uPAR PET SUVmax = 2.9.

**Figure 3:**
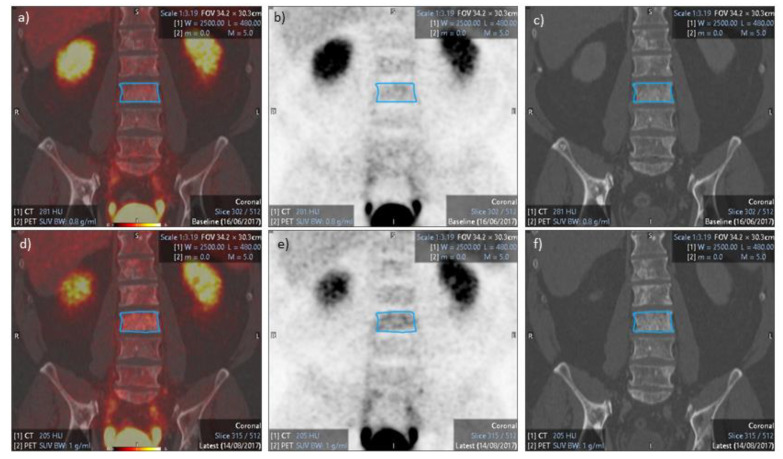
uPAR PET/CT (patient 10) Coronal fused images (**left**), PET images (**centre**) and CT images (**right**). Baseline images (**a**–**c**) and follow-up after two cycles of ^223^RaCl_2_ (**d**–**f**). Bone metastasis in lumbar spine (L3) is delineated, baseline SUVmax was 2.4 and after two cycles of ^223^RaCl_2_ increased to 3.9. After end of therapy the patient was diagnosed with epidural involvement at L3 and received EBRT to relieve symptoms.

The mean SUV_max_ of all lesions for both observers was 2.10 (Range: 0.70–5.00) at baseline and 2.20 (Range: 0.82–4.00) at follow-up (examples in Figure 2 and Figure 3). In normal liver tissue mean SUV_max_ was 1.87 (SD 0.40) at baseline and 1.88 (SD: 0.16) at follow-up. Mean SUV_max_ in the psoas muscle and thoracic aorta was 1.05 (SD 0.21)/1.17 (SD: 0.27) and 3.51 (SD: 0.60)/3.44 (SD: 0.41) at baseline/follow-up.

### 3.2. Overall Survival

**Figure 4:**
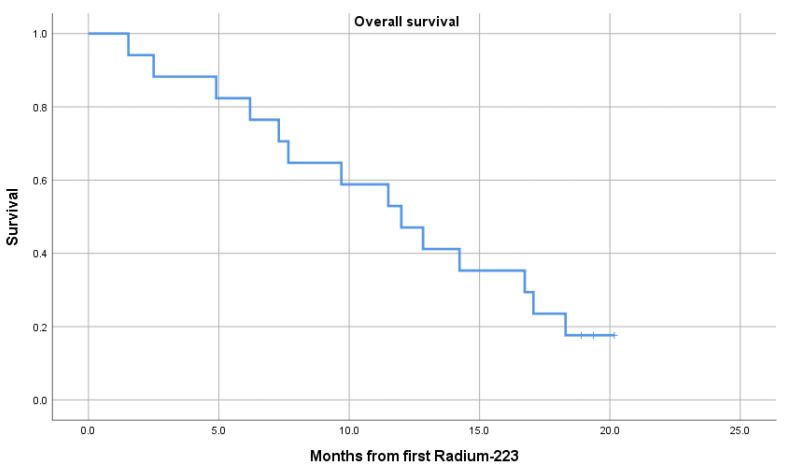
Kaplan-Meier curve of overall survival. Median OS 12 mo. (95% CI: 7.8–16.2).

Mean OS was 11.9 months (95% CI: 9.1–14.8) from the first cycle of ^223^RaCl_2_. For survival curve and median OS, please see Figure 4. There was a significant association between baseline SUV_max_ of the index lesion and OS with a hazard ratio of 2.51 (95% CI: 1.01–6.28, *p* = 0.05) per unit increase in SUV_max_. Cox regression analysis for testing the association between mean SUV_max_ of all lesions at baseline and OS only reached borderline statistical significance (Hazard ratio 3.36, 95% CI: 0.9–12.7, *p* = 0.07). No significant association between change in SUV_max_ from baseline to follow-up and OS was found.

### 3.3. Response

Eight patients (47%) experienced radiographic progression during therapy; four patients were diagnosed with progression in soft tissue, two patients with skeletal progression and two patients were classified as progressing in both bone and soft tissue. Median rPFS was 4.8 months (95% CI: 1.8–7.8). There was no significant association between SUVmax of the index lesion (baseline value as well as change during therapy) and radiographic progression during therapy or rPFS.

### 3.4. Symptomatic Skeletal Events

**Figure 5:**
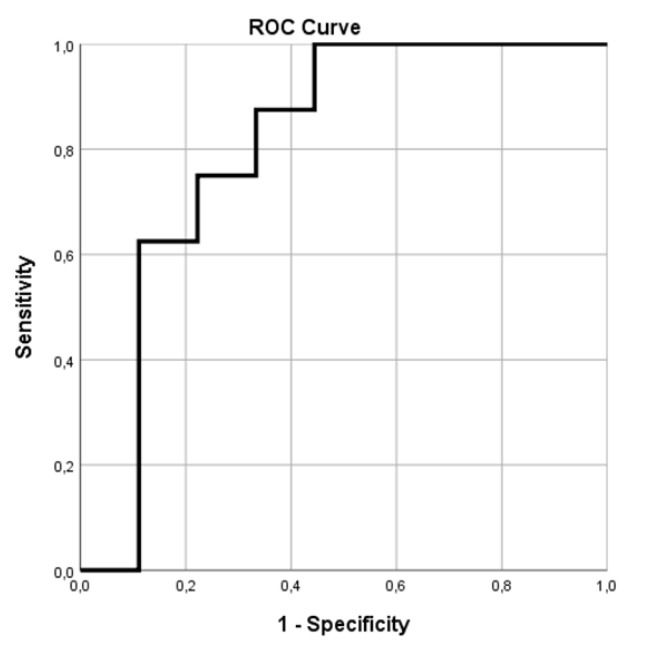
ROC curve analysis of SUVmax of the index lesion in predicting occurrence of SSE. AUC = 0.81 (95% CI: 0.58–1.00, *p* = 0.034).

During the first 12 months after initiation of ^223^RaCl_2_-therapy, eight patients experienced a SSE (six patients with EBRT (35% of total), while there was one case of pathological symptomatic fracture (6%) and one patient with spinal cord compression (6%). ROC analysis of baseline SUV_max_ of the index lesion as predictor of SSE had an area under the curve (AUC) = 0.81 (95% CI: 0.58–1.00, *p* = 0.034) (Figure 5). Based on the ROC analysis, a cutoff for obtaining the highest accuracy of SUV_max_ = 2.34 was suggested. Patients with SUV_max_ at baseline above this cutoff value had odds ratio = 14.0 (95% CI: 1.14–172.6, *p* = 0.023) for occurrence of SSE within 12 months. No significant association between changes in SUV_max_ during therapy and the risk of SSE was found.

## 4. Discussion

Assessment of efficacy is important in all oncological treatments to avoid futile therapy with potential side-effects. In the case of ^223^RaCl_2_ therapy, there is no clear consensus regarding the role of imaging in the evaluation of interim response to therapy [16,17]. Although bone scintigraphy and ^18^F-fluoride PET are the companion imaging agents of the bone targeted ^223^RaCl_2_ therapy, these modalities are not suitable for early response assessment. As increased osteoblastic activity in new and existing lesions can reflect effect of treatment as well as progression, response to ^223^RaCl_2_ cannot be evaluated before end of therapy [18]. This leads to a substantial risk that patients who do not benefit from ^223^RaCl_2_ therapy may continue ineffective treatment undetected.

In this phase II study, we wanted to assess whether uPAR PET/CT could serve as a method of early response assessment in patients with mCRPC treated with ^223^RaCl_2_. We hypothesized that monitoring change in uPAR expression could provide an alternative approach to response assessment by imaging the invasive potential of the disease rather than bone remodeling. Although based on a small number of patients, SUV_max_ at baseline was significantly associated with survival and risk of SSE, thus indicating that the aggressiveness of prostate cancer lesions can be determined non-invasively by uPAR PET.

The small number of patients in the study was due to challenges in recruitment. As displayed in Table 1, the majority of participants had advanced disease with high PSA and extent of disease, which is representative of the patient cohort receiving ^223^RaCl_2_ at our institution. These patients are physically and mentally affected by their illness, and therefore more prone to decline participation in studies involving additional visits to the hospital. Furthermore, results from the ERA-223 trial [19], subsequent changes in guidelines regarding indication for ^223^RaCl_2_ therapy [20] as well as other emerging therapies for mCRPC becoming available, all led to a significant reduction in the number of patients eligible for inclusion. If it had been possible, more patients would have been advantageous to allow the addition of other variables in the prediction models and achieve more robust results.

Sensitivity in detection of metastases was not an endpoint of the study, and as the examples in Figure 2 and Figure 3 show, the tumor-to-background ratio was not optimal for visualization of metastases. In fact, the selection of the bone lesions for quantitative analysis of uPAR ligand uptake was based upon bone scintigraphy. This represents a challenge in using uPAR PET/CT for staging or response assessment in mCRPC, especially in detection of new metastases. Additionally, the absolute changes in SUV_max_ in bone metastases after two cycles of ^223^RaCl_2_ were in most cases very discrete and were not associated with progression after end of therapy, which implies that uPAR PET should probably not be used for assessing response in this setting.

The reason for the relatively low uptake of uPAR ligand in bone metastases is yet to be determined. The binding peptide AE105 as well as the chelated form NOTA-AE105 are antagonists with high affinity for uPAR as validated in several studies [13,21,22,23]. Consequently, if the uPA/uPAR system was highly activated in the bone lesions, one might expect a more marked uptake. Nevertheless, the finding of this study of a clear association between avidity on uPAR PET and OS, strongly supports that uPAR PET does reflect the uPAR expression. However, in the present study, it was not possible to obtain biopsies from bone metastases to directly validate the expression of uPAR. It has been demonstrated that the uPA/uPAR system plays an important part in osseous metastatic dissemination in prostate cancer. Accordingly, blocking the uPAR signaling with either anti-uPAR antibody or antisense oligonucleotides reduced development of bone metastases in mice inoculated with prostate cancer cells [8,24]. Likewise, uPA-silenced prostate cancer cells inoculated in xenograft bone in mice exhibited decreased growth and tumor size [25]. Therefore, the relatively moderate uptake of ^68^Ga-NOTA-AE105 implies that, although the uPA/uPAR system is active in the formation of new bone lesions, the expression may shift and be lower in existing bone metastases.

The challenge of identifying metastases due to low tumor-to-backgound ratio can be circumvented by combining uPAR PET with anatomically detailed imaging as magnetic resonance imaging (MRI) or with bone scintigrapy (SPECT or PET).

Other imaging modalities for assessment of response to ^223^RaCl_2_ have been proposed [18]. An appealing option in this setting is ^68^Ga-PSMA PET/CT [26] which, as opposed to bone targeted imaging, can detect metastatic disease in both bone and soft tissue. Additionally, interim response assessment is not distorted by the flare phenomenon as seen with bone targeted imaging. Recently, a proposal for PSMA PET response assessment in systemic therapy has been published [27], but is yet to be validated prospectively.

## 5. Conclusions

In conclusion, this phase II study found that the non-invasive quantitative evaluation of uPAR expression by uPAR-PET using ^68^Ga-NOTA-AE105 can provide prognostic information regarding OS and risk of SSE in patients with advanced mCRPC receiving ^223^RaCl_2_. A relatively low uptake of the uPAR ligand in bone metastasis impedes direct visual evaluation and requires another modality, e.g., bone scintigraphy or MRI, for lesion delineation. The change in ^68^Ga-NOTA-AE105 uptake after two cycles of ^223^RaCl_2_ therapy was not associated with disease progression. 

## Figures and Tables

**Table 1:** Patient characteristics at initiation of ^223^RaCl_2._

Pt. No.	Age (Years)	ECOG Performance Status	Number of Bone Metastases ^†^	s-PSA (µg/L)	s-ALP ^†^ (U/L)	Previous Systemic Therapies for mCRPC
1	76	1	>20	931	NA	Doce, Caba, Enza, Abi
2	73	0	6–20	26	66	Doce
3	76	1	>20	7	82	Doce, Enza
4	78	1	>20	494	231	Doce, Caba, Enza, Abi
5	69	1	>20	318	99	Doce, Enza
6	81	1	6–20	109	73	Doce, Caba, Abi
7	63	0	<6	5	69	Doce
8	69	1	>20	235	271	Doce, Caba
9	76	1	>20	68	507	Doce, Caba
10	71	1	>20	52	350	Doce
11	82	0	>20	87	75	Enza, Abi
12	76	0	>20	119	85	Doce, Abi
13	78	1	>20	74	265	Doce, Enza
14	76	1	>20	942	108	Doce, Caba, Enza, Abi
15	79	1	>20	99	77	Enza, Abi
16	83	1	>20	77	98	Doce, Enza
17	77	1	6–20	113	140	Doce, Enza

PSA = Prostate specific antigen, ALP = Alkaline Phosphatase, Doce = Docetaxel, Caba = Cabazitaxel, Enza = Enzalutamide, Abi = Abiraterone. NA = Not assessed. ^†^ Number of bone metastases graded according to Soloway [15].

**Table 2:** ^68^Ga-NOTA-AE105 uptake in lesions and clinical outcomes.

Pt. No.	SUV_max_ ^ Baseline	SUV_max_ ^ 2 Cycles	ΔSUV_max_	No. of ^223^Ra Cycles	ΔPSA from Baseline to EOT (%)	ΔALP from Baseline to EOT (%)	Radiographic Progression during Therapy	SSE (12 mo. from First ^223^Ra)	OS (mo. from First ^223^Ra)	Follow-Up (mo. from First ^223^Ra)
1	3.92	NA *	NA *	1	NA	NA	NA	-	1.5	
2	2.09	1.65	−0.44	6	246.2	18.2	-	-		19.4
3	2.89	3.10	0.21	3	47.0	-23.2	Soft tissue	EBRT		18.9
4	1.90	NA *	NA *	1	NA	NA	Soft tissue	-	9.7	
5	2.53	2.45	−0.08	3	73.0	55.6	-	EBRT	14.2	
6	2.79	2.86	0.07	3	166.1	46.6	Bone	EBRT	11.5	
7	1.22	1.39	0.17	6	269.6	−13.0	-	-		20.2
8	2.11	2.55	0.44	5	86.0	21.0	Bone	-	7.3	
9	2.39	NA *	NA *	1	NA	NA	Soft tissue	Fracture	4.9	
10	2.79	2.92	0.13	4	73.1	−64.3	-	EBRT	16.7	
11	1.67	1.70	0.03	6	34.5	−60.0	-	-	17.1	
12	3.82	2.75	−1.07	3	106.7	−16.5	-	EBRT	7.7	
13	2.29	3.23	0.95	4	604.1	−30.9	-	-	12.0	
14	2.43	2.77	0.34	2	30.5	25.9	Soft tissue	-	2.5	
15	2.22	2.35	0.13	5	796.0	94.8	Bone + Soft tissue	Spinal cord compression	12.8	
16	3.52	3.11	−0.41	3	10.8	28.6	Bone + Soft tissue	EBRT	6.2	
17	2.59	2.93	0.35	6	252.2	−40.7	-	-	18.3	

^ SUV_max_ of the index lesion (mean of the two observers). * Patients who completed <2 cycles of ^223^RaCl_2_ did not undergo the follow-up uPAR PET/CT.

## Data Availability

The data presented in this study are available on request from the corresponding author. The data are not publicly available due to restrictions by the Danish Agency for Data Protecion.
